# Unusual respiratory capacity and nitrogen metabolism in a Parcubacterium (OD1) of the Candidate Phyla Radiation

**DOI:** 10.1038/srep40101

**Published:** 2017-01-09

**Authors:** Cindy J. Castelle, Christopher T. Brown, Brian C. Thomas, Kenneth H. Williams, Jillian F. Banfield

**Affiliations:** 1 Department of Earth and Planetary Sciences, University of California, Berkeley, California, United States; 2 Department of Plant and Microbial Biology, University of California, Berkeley, California, United States; 3 Earth and Environmental Sciences Division, Lawrence Berkeley National Laboratory, Berkeley, California, United States

## Abstract

The Candidate Phyla Radiation (CPR) is a large group of bacteria, the scale of which approaches that of all other bacteria. CPR organisms are inferred to depend on other community members for many basic cellular building blocks and all appear to be obligate anaerobes. To date, there has been no evidence for any significant respiratory capacity in an organism from this radiation. Here we report a curated draft genome for ‘Candidatus *Parcunitrobacter nitroensis*’ a member of the Parcubacteria (OD1) superphylum of the CPR. The genome encodes versatile energy pathways, including fermentative and respiratory capacities, nitrogen and fatty acid metabolism, as well as the first complete electron transport chain described for a member of the CPR. The sequences of all of these enzymes are highly divergent from sequences found in other organisms, suggesting that these capacities were not recently acquired from non-CPR organisms. Although the wide respiration-based repertoire points to a different lifestyle compared to other CPR bacteria, we predict similar obligate dependence on other organisms or the microbial community. The results substantially expand the known metabolic potential of CPR bacteria, although sequence comparisons indicate that these capacities are very rare in members of this radiation.

The Candidate Superphylum OD1 bacteria, also known as Parcubacteria, were first identified by phylogenetic analysis of 16S rRNA genes recovered from environmental samples^1^. To date, there are no isolated representatives, but symbiotic lifestyles have been inferred from genomic analyses based on the lack of genes for biosynthesis of most amino acids, nucleotides, vitamins and lipids. The environments from which these bacteria were sampled have mostly been anoxic^1^,^2^,^3^,^4^,^5^,^6^,^7^,^8^,^9^,^10^,^11^,^12^. As of today, metabolic predictions for the members of the Parcubacteria support anaerobic fermentative metabolisms^3^,^6^,^8^,^9^,^12^,^13^, and some likely also impact hydrogen and sulfur cycles^6^,^7^. Members of the Parcubacteria superphylum along with other CPR members were inferred to be non-respiring due to the lack of most genes for electron transport chain components and the tricarboxylic acid cycle (TCA). Based on these metabolic analysis, it was suggested that Parcubacteria obligately ferment sugars to organic acids, although some are apparently capable of degrading complex carbon^6^,^7^,^9^. Interestingly, however, some Parcubacteria have been identified in oxic groundwater and several of the genomes encoded for cytochrome o ubiquinol oxidase (Complex IV)^12^,^13^. Complex IV is involved in aerobic metabolism (reducing O_2_ to H_2_O) and its presence in Parcubacteria was striking. However, the lack of other electron transport components pointed toward a function in O_2_ scavenging rather than in energy conservation^12^.

In 2011 we sampled microbial communities from an aquifer adjacent to the Colorado River in Rifle, Colorado, USA^8^,^12^,^14^. A time series of anoxic groundwater samples was collected prior to (time point A) and during (B–E) an acetate biostimulation experiment, and included samples taken after acetate addition ceased (F). Biomass was collected for metagenome sequencing at each time point using 0.1 and 0.2-μm filters after groundwater was passed through a 1.2-μm pre-filter. This strategy was designed specifically to target small cells, which were predicted to be abundant based on enrichment of organisms (including CPR) with small genomes in previous acetate amendment experiments^8^,^11^. Here, we report a detailed analysis of a previously reported genome for a bacterium associated with the Parcubacteria superphylum based on phylogenetic analyses of ribosomal protein and 16S rRNA gene sequences^15^. The genome was reconstructed from a metagenome obtained by sequencing DNA from cells collected from groundwater on the 0.2 μm filter prior to acetate amendment^12^. This genome was selected based on identification of genes that suggested the existence of respiratory capacity, a feature not reported to date within the CPR, despite availability of over a thousand genomes^12^,^16^.

## Results and Discussion

### Genome binning and curation

**Figure 1:**
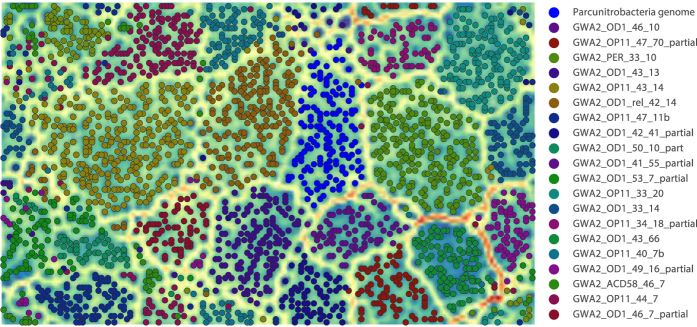
Validation of the Parcunitrobacteria draft genome using tetranucleotide frequencies and time-series abundance patterns. Tetranucleotide frequencies and coverage were determined over 5 Kbp non-overlapping sliding windows for the Parcunitrobacteria genome along with a subset of genome bins from the GWA2 metagenome. The data were normalized and the ESOM was trained for 10 epochs using the Somoclu algorithm (https://arxiv.org/abs/1305.1422) using the option to initialize the codebook using PCA. Boundaries (dark bands) separate clusters of fragments with similar signatures (each dot represents a 5 kb fragment). The map was colored based on the binning information.

We further manually curated a draft genome previously reconstructed from the groundwater sample collected prior to acetate injection into the aquifer (sample GWA2)^12^,^14^. In the current study, the binning was confirmed by emergent self-organizing map (ESOM) analysis of tetranucleotide sequence composition as well as time series abundance information (Fig. 1). A few scaffolds (likely phage) and part of a chimeric scaffold were removed, and other scaffolds added based on these analyses. In addition, the genome was subjected to further assembly curation. The final, revised genome is deposited at NCBI (LBUF00000000). The genome was curated into 18 scaffolds with a total length of 0.884 Mb. The genome assembly resulted in depths of coverage of 13x and has an average GC content of 37.8%, and 886 predicted protein-coding sequences. We recovered single copies of the 23S rRNA and 16S rRNA genes and identified three small insertion sequences in the 16S rRNA gene (157–214 nucleotides in length). Transcriptomic sequencing data indicates that these insertions are spliced out of the RNA sequence, as occurs with many other CPR bacteria^12^. We estimate that the genome very close to 100% complete, based on detection of 43 universal single copy genes (SCG) determined to be appropriate for analysis of CPR genomes^12^.

### The bacterium is a member of the Parcubacteria superphylum

**Figure 2:**
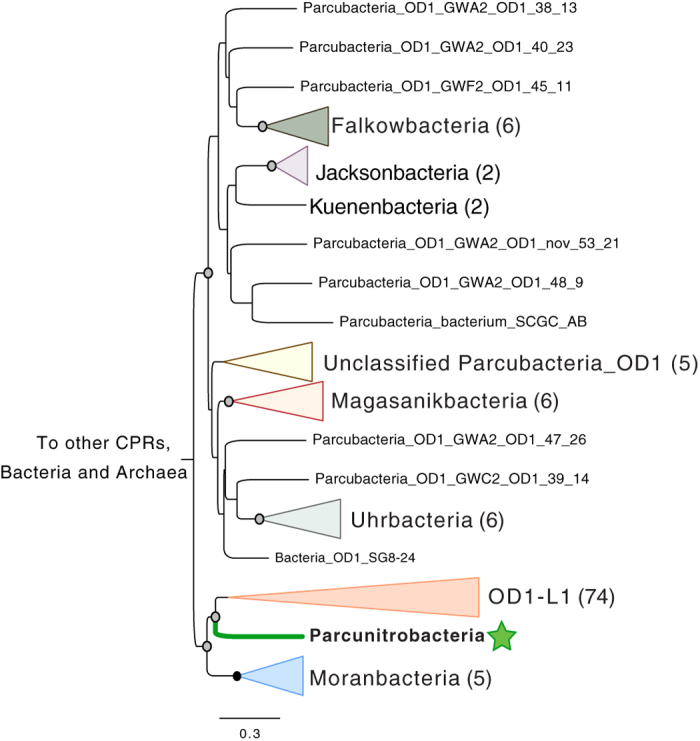
Phylogenetic analyses places the Parcunitrobacteria within the OD1 superphylum. Maximum-likelihood phylogeny of the 16S rRNA gene places the organism adjacent to the OD1-L1 group, possibly in a new phylum-level lineage. The phylogeny was inferred using RAxML-HPC under the GTRCAT model of evolution with 100 bootstrap re-samplings. Bootstrap support values are indicated by circles on nodes; black for support of 100%, grey for support from 50 to 99%. The complete 16S tree is available in Newick format in Supplementary Dataset 1.

Phylogenetic analyses of the 16S rRNA gene of the bacterium studied here along with previously reported sequences from CPR genomes^12^,^15^ and clone sequences identified in the SILVA database^17^ confirmed that this organism falls within the Parcubacteria superphylum. The 16S rRNA gene sequence shares 83% identity with the closest sequence in NCBI, which is from an uncultivated clone (TANB108) from TCE-dechlorinating groundwater^18^. In a phylogenetic tree, the 16S rRNA sequence places next to a large group of >90 phyla. This group was named OD1-L1 because ribosomal protein L1 (rpL1) is lacking from all organisms in this group^12^. The genome analyzed here is also missing the gene for rpL1. Given this, and the fact that a recently published concatenated ribosomal protein tree places this organism within the OD1-L1^15^, it may represent a new phylum-level lineage within that group (Fig. 2). Loss of rpL1 results in severe growth defects^19^. Because of the role of this protein in ribosome biogenesis, it was suggested that OD1-L1 CPR bacteria may have alternative mechanisms for ribosome regulation in addition to having unusual ribosome structures^12^. As for all CPR and TM6 bacteria, the genome studied here also lacks ribosomal protein L30, a ribosomal protein that has been shown to be non-essential for some organisms when grown under laboratory conditions^12^,^19^.

Given indications of novel metabolic potential, we confirmed the classification of important individual scaffolds as deriving from a Parcubacteria bacterium by phylogenetic and sequence similarity analyses. Sequence-similarity analysis involved comparison between genes on those scaffolds and genes in other Parcubacteria genomes. The correct assembly of a key scaffold was confirmed by direct visualization of paired read mapping. We investigated, in detail the functional predictions for key enzymes encoded in the genome, and predictions were refined based on phylogenetic information, detailed active site analysis, and protein structure prediction analysis.

### Energy Metabolism

Metabolic predictions indicate that the organism has the genomic potential for utilizing a variety of organic compounds (including ribose, glucose, acetate and possibly propionate) as energy and carbon sources. It has 50% of the oxidative tricarboxylic acid (TCA) cycle, a near-complete glycolysis pathway and an oxidative phosphorylation pathway. The TCA cycle of this bacterium includes a putative 2-oxoacid-ferredoxin oxidoreductase (which may have a broader substrate range, including pyruvate), fumarate hydratase, a type B succinate dehydrogenase/fumarate reductase (SDH/FRD) that catalyzes the reversible conversion of fumarate to succinate^20^,^21^. The genome also encodes a putative citrate synthase annotated as citrate synthase/methylcitrate synthase. It has been shown that the second citrate synthase (CS) gene encoded by the genome of *Escherichia coli* is in fact a 2-methylcitrate synthase that has minor citrate synthase activity^22^. As the genome encodes only one copy of the CS, we postulate that this enzyme may have dual function. Whereas, citrate synthase is constitutively produced in *E. coli*, 2-methylcitrate synthase is induced during growth on propionate.

**Figure 3:**
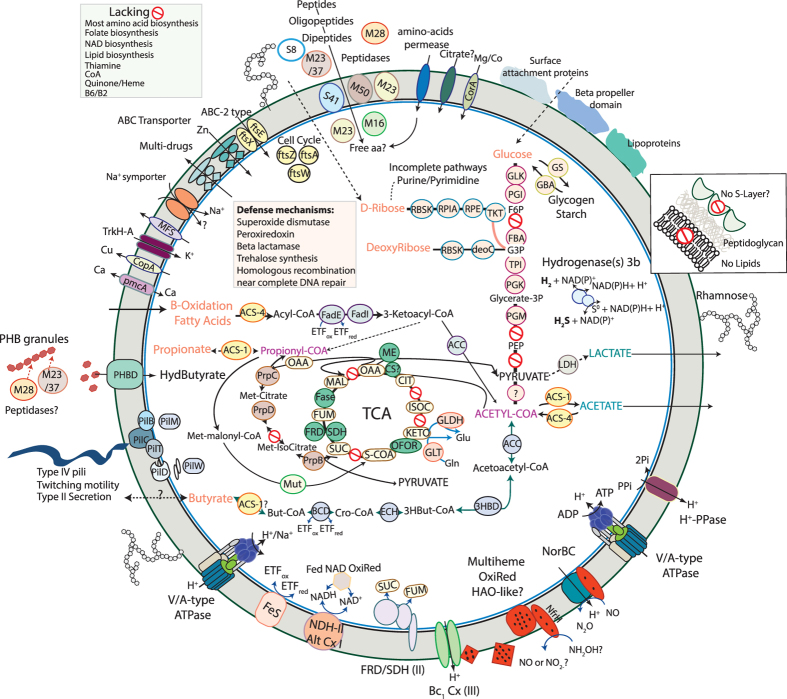
Energy metabolism of ‘Candidatus *Parcunitrobacter nitroensis*’. Red “no entry” signs indicate genes missing from pathways. Abbreviations not defined in the text: TCA, tricarboxylic acid cycle; Glk, glucokinase; Pgi, glucose-6- phosphate isomerase; FBP, fructose-1,6-bisphosphatase; Pfk, phosphofructokinase; Aldo, fructose-bisphosphate aldolase; Pgk, phosphoglycerate kinase; TPI, triosephosphate isomerase; PHBD, polyhydroxybutyrate depolymerase; PGM, 1,3-bisphosphoglycerate-independent phosphoglycerate mutase; PPi, pyrophosphate; Pi, inorganic phosphate; OFOR, oxoglutarate/2-oxoacid-ferredoxin oxidoreductase; ACS, acetyl-CoA synthetase; LDH, lactate dehydrogenase; ME, malate dehydrogenase (oxaloacetate-decarboxylating); GS, glycogen synthase; GBA, glucosidase; RBKS, ribokinase; RpiA, ribose 5-phosphate isomerase A; RPE, ribulose-phosphate 3-epimerase; Tkt, transkelotase; deoC, deoxyribose-phosphate aldolase; GLDH, glutamate dehydrogenase; GLT, glutamate synthase (NADPH); Mut, methylmalonyl-CoA mutase;_rpC, 2-methylcitrate synthase; PrpD, 2-methylcitrate dehydratase; PrpB, methylisocitrate lyase; FadE, Acyl-CoA Dehydrogenase; FadJ, 3-hydroxyacyl-CoA dehydrogenase, enoyl-CoA hydratase; ACC, acetyl-CoA acetyltransferase or 3-ketoacyl-CoA thiolase; ETF, electron transfer flavoprotein; FeS, Electron-transferring-flavoprotein dehydrogenase/ubiquinone oxidoreductase; BCD, butyryl-CoA dehydrogenase; ECH, enoyl-CoA hydratase/isomerase; 3HBD, 3-hydroxybutyryl-CoA dehydrogenase; H+-PPase, proton-translocating pyrophosphatase; ATPase, ATP synthase; Cx I, NADH dehydrogenase; Cx II, succinate dehydrogenase/fumarate reductase; Fase, fumarate hydratase; CS, citrate synthase Cx III, bc1 complex; NorBC, nitric-oxide reductase; HOA, hydroxylamine oxidoreductase. The major classes of peptidases include: M50, M23, M16, M28, M23/37, S8 and S41 as presented in the figure. All of the protein sequences can be accessed at http://ggkbase.berkeley.edu/genome_summaries/1088-Parcunitrobacteria_curated_genome.

Multiple genes involved in propionate oxidation are co-located in the genome. Indeed, we identified the following enzymes of the 2-methylcitrate cycle: 2-methylcitrate synthase (PrpC), 2-methylcitrate dehydratase (PrpD) and methylisocitrate lyase (prpB). The 2-methylcitrate cycle is an important pathway in which propionate is oxidized to pyruvate and succinate^23^. In *E. coli*, and *Salmonella enterica*, genes for propionate breakdown are co-located (*prpBCDE*) and are, with the exception of *prpE* (propionyl-CoA synthetase), essential for growth on propionate as the sole carbon and energy source^23^,^24^. The gene *prpE* that encodes propionyl-CoA synthetase, which mediates the activation of propionate to propionyl-coenzyme A (CoA)^25^, was not identified in the organism studied here. However, it may be capable of utilizing an ADP-forming acetyl-CoA synthetase encoded in the genome to activate propionate^26^; Fig. 3). The last step of this cycle involving the methylisocitrate lyase produces pyruvate that may be further oxidized to acetyl-CoA or used as a building block for biosynthesis.

Another striking finding of the genome is the presence of a methylmalonyl-CoA mutase, which is involved in the isomerization of methylmalonyl-CoA to succinyl-CoA. Methylmalonyl-CoA is likely derived from propionyl-CoA and it is involved in key metabolic pathways including the oxidation of propionate via the methylmalonyl-CoA pathway. This cycle is an alternative pathway for breakdown of propionate but depends on the availability of vitamin B12 as a cofactor for functionality of its key enzyme, the methylmalonyl-CoA mutase^27^. It appears that this Parcubacterium is unable to synthetize vitamin B12, suggesting that this essential cofactor must be acquired from other sources. The gene encoding the mutase is located near the genes from the methylcitrate cycle. However, the two other key enzymes of this pathway, propionyl-CoA carboxylase and methylmalonyl-CoA epimerase, which convert propionyl-CoA to methylmalonyl-CoA, were not identified. These functions may be conferred by some of the many genes of unknown function or genes that were missed due to gaps in the genome. In view of the numerous enzymes involved in propionate metabolism, we hypothesize that this short-chain fatty acid may be a carbon source for this bacterium, yet some aspects of the mechanism remain to be discovered.

The genome encodes enzymatic machinery for short chain fatty acid metabolism. Unexpectedly (given that this capacity has not previously been reported in the CPR), this bacterium metabolizes fatty acids by the ß-oxidation pathway, for which genes for all of the ß-oxidation enzymes were identified (Fig. 3). Even more surprisingly, there are multiple homologues for the entire ß-oxidation pathway that are co-localized in the genome. Specifically, the genome contains two acyl-CoA dehydrogenase genes, two enoyl-CoA hydratase genes, one 3-hydroxybutyryl-CoA dehydrogenase gene, two acetyl-CoA acetyltransferase genes, one acyl-CoA synthetase (AMP-forming), and one acetyl-CoA synthetase (ADP-forming). The single enzyme variant acetyl-CoA synthetase (ADP-forming) is involved in acetate formation and energy conservation whereas the (AMP)-forming acetyl-CoA synthetase is the key enzyme for converting acetate to acetyl-CoA. Its substrate range may include propionate, butyrate or other short-chain fatty acids^26^. The sequences of all of these enzymes associated to fatty acids metabolism are highly divergent from sequences found in other organisms, suggesting that these capacities were not recently acquired from non-CPR organisms (Table S1).

**Figure 4:**
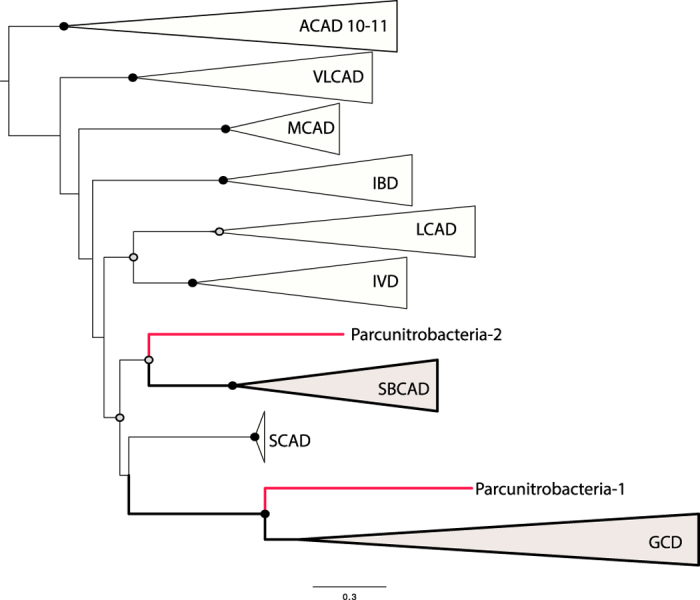
Maximum-likelihood phylogeny of acyl-CoA dehydrogenase protein family. The phylogeny was estimated from 63 sequences using PHYML^69^. Abbreviations: ACAD, Acyl-CoA dehydrogenases; VLCAD, Very long-chain acyl-CoA dehydrogenase; MCAD, Medium-chain acyl-CoA dehydrogenase, IBD, Isobutyryl-CoA dehydrogenase; LCAD, Long-chain acyl-CoA dehydrogenase; IVD, Isovaleryl-CoA dehydrogenase; SBCAD, Short/branched-chain acyl-CoA dehydrogenase, also known as 2-methyl branched chain acyl-CoA dehydrogenase; GCD, Glutaryl-CoA dehydrogenase. Bootstrap support values are indicated by circles on nodes; black for support of 100%, grey for support from 50 to 99%. The complete acyl-CoA dehydrogenase protein family tree is available in Newick format in Supplementary Dataset 2.

In order to more completely characterize the acyl-CoA dehydrogenases identified here, we conducted a phylogenetic analysis of this large family, as substrate specificity is the primary characteristic used to define its members. Phylogenetic analysis (Fig. 4) reveals that the two genes encoding the acyl-CoA dehydrogenases cluster within two distinct groups. One belongs to the subfamily of glutaryl-CoA dehydrogenase (GDH^28^,^29^) and the other falls into the short/branched-chain acyl-CoA dehydrogenase subfamily, also known as 2-methyl branched-chain acyl-CoA dehydrogenase (SBCAD). In addition to their function in β-oxidation of glutaryl-CoA to crotonyl-CoA, GDHs play a role in the catabolism of lysine and tryptophan in many organisms. In anaerobic bacteria that degrade aromatic compounds GDHs play an essential role in the benzoyl-CoA degradation pathway.

Genes involved in benzoate metabolism as well as in lysine and tryptophan degradation were not identified in the genome, thus the source of glutaryl-CoA is unclear. The second acyl-CoA dehydrogenase identified as SBCAD might act as butyryl-CoA dehydrogenase, based on biochemical evidence^30^ and sequence analysis confirms that this gene might be involved in butyrate fermentation (or production). If producing butyrate, acetyl-CoA may be converted to butyryl-CoA by the action of four enzymes encoded in the genome studied here: acetyl-CoA acetyltransferase, 3-hydroxybutyryl-CoA dehydrogenase, putative 3-hydroxybutyryl-CoA dehydratase, acyl-CoA dehydrogenase (SBCAD), and the electron transfer flavoproteins (ETF) that serve as electron acceptor for numerous acyl-CoA dehydrogenases (Fig. 3). Interestingly, the genes encoding for ß-oxidation of fatty acids along with the ETF are co-located in the genome, which strongly suggests their involvement in the same metabolic pathway. We did not identify the enzyme involved in converting butyryl-CoA to butyrate but it may be capable of utilizing the identified ADP-forming acetyl-CoA synthetase to produce butyrate and generate ATP, analogous to *Pyrococcus* spp.^31^,^32^.

Lacking are genes required for gluconeogenesis (fructose-1,6-bisphosphatase, pyruvate carboxylase and PEP carboxykinase). We predict that this bacterium might be capable of sugar fermentation to produce ATP, coupled to acetate or butyrate production. Sugars may be degraded by the upper EMP glycolytic pathway with a compensation of the enzymes of the Pentose Phosphate pathway for the lack of phosphofructokinase, as previously suggested for other CPR bacteria^6^,^33^. Several enzymes, including enolase and pyruvate kinase of the lower glycolytic pathway are lacking, thus the capacity to produce pyruvate is unclear. However, an oxoglutatarate/2-oxoacid-ferredoxin oxidoreductase is present in the genome and may convert pyruvate to acetyl-CoA. The organism is also inferred to be able to degrade ribose to 3-phosphoglycerate that feeds into the lower arm of glycolysis. Subsequently it would lead to acetyl-CoA, which may be converted to acetate or butyrate, with the concomitant production of ATP as described above. However, as two key enzymes of the lower glycolytic pathway are lacking, the mechanism for conversion of sugar to acetyl-CoA is unclear.

Another potential carbon and energy source is polyhydroxybutyrate (PHB), a storage compound produced by many bacteria under imbalanced growth conditions, such as when there is an excess in carbon but limited availability of nitrogen, phosphorus, magnesium, oxygen or sulfur compounds^34^. After bacterial cell death and lysis, PHB granules may be released to the environment where they could potentially serve as a carbon source for other microorganisms. The genome encodes a PHB depolymerase and several peptidases predicted to be extracellular. Thus, we hypothesize that extracellular and cell wall peptidases break down the protein shell of the PHB granules, releasing the PHB molecule, which could then be degraded by the PHB depolymerase into β-hydroxybutyrate, which may be used as carbon and energy sources. Specifically, an encoded 3-hydroxybutyryl-CoA dehydrogenase could oxidize β-hydroxybutyrate to acetoacetate that may then be activated by acyl-CoA synthase to form acetoacetyl-CoA. The concerted actions of acetyl-CoA C-acetyltransferase and acetyl-CoA synthetase convert acetoacetyl-CoA to acetate with the concomitant production of ATP.

The organism may be capable of synthesizing and/or utilizing common energy-storage polysaccharides, as we identified several genes encoding enzymes for starch or glycogen metabolism (Fig. 3). This capacity has been previously described in the Peregrinibacteria phylum of the CPR^33^.

**Figure 5:**
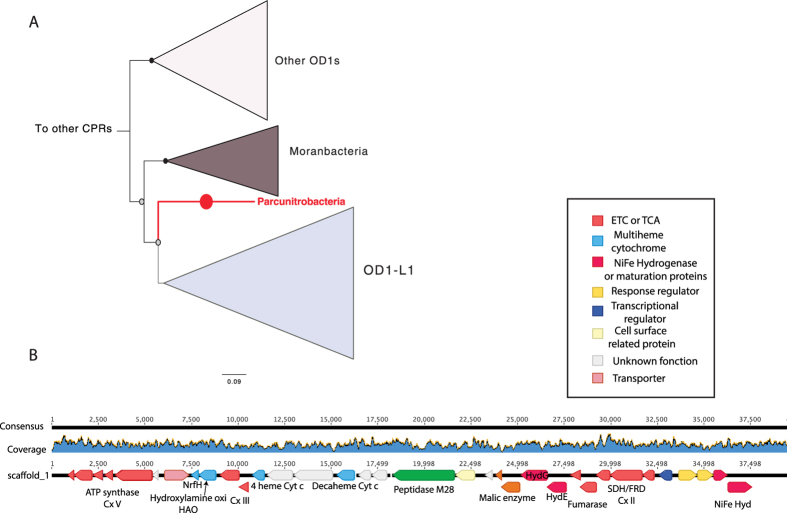
(**A**) DNA gyrase subunit A maximum-likelihood phylogeny (the gene is located on the GWA2_OD1-rel_38_13_curated_scaffold_1) showing placement of Parcunitrobacteria within the Parcubacteria superphylum; the complete gyrA protein tree is available in Newick format in Supplementary Dataset 3. (**B**) Genome sequence coverage, gene synteny, and annotation of the GWA2_OD1-rel_38_13_curated_scaffold_1 where most of the genes encoding for respiratory capacities were detected.

Remarkably, this is the first member of the bacterial Candidate Phyla Radiation reported to have a genome that encodes a complete oxidative phosphorylation pathway. The pathway includes one single-unit type-2 NADH dehydrogenase complex (alternative Complex I^35^, a succinate dehydrogenase/fumarate reductase (Complex II; SDH/FRD), a PETAB complex (complex III; cytochrome *b*-Rieske type complex), a nitric-oxide (NO) reductase (belonging to the family of heme-copper oxidase or Complex IV) and one V/A-type ATPase (Complex V). Interestingly, most of these genes involved in respiration are co-located on the same scaffold (GWA2_OD1-rel_38_13_curated_scaffold_1). We conducted a phylogenetic analysis of the gene *gyrA* that was co-encoded on this scaffold to confirm the assignment of this critical scaffold to the genome, and also verified the accuracy of the scaffold assembly by inspection of paired read sequences (Fig. 5A and B).

Co-localized on GWA2_OD1-rel_38_13_curated_scaffold_1 are genes for one ATPase, putative hydroxylamine oxidoreductase (HAO; 9 hemes *c*), PETAB complex, SDH/FRD as well as one type 3b hydrogenase. Also in close proximity we identified three additional multiheme *c*-type cytochromes (one decaheme and two tetrahemes) that are essential electron carriers in respiration. One tetraheme cytochrome gene is found next to the genes encoding for the PetAB complex, suggesting that it might form a *bc*_*1*_ complex (III) (complex III). Consistent with this function, this multiheme cytochrome is predicted to be membrane-bound, as is the typical monoheme cytochrome *c* subunit of this complex III. The second tetraheme cytochrome, co-located next to the HAO gene, is a member of the NapC/NrfH family, which consists of multiheme *c*-type cytochromes that exchange electrons with oxidoreductases^36^. They form a group of membrane-bound quinol dehydrogenases that are essential components of several electron transport chains, including those involved in nitrogen metabolism. Based on studies of *Nitrosomonas europaea*, hydroxylamine oxidation is assumed to proceed through catalysis by the octaheme HAO followed by electron transfer to membrane quinone mediated by a first tetraheme cytochrome and the membrane-associated tetraheme cytochrome. The latter channels electrons into the electron transport chain to terminal oxidases^37^. It has been shown that HAO may catalyze the oxidation of hydroxylamine (NH_2_OH) to nitric oxide (NO)^38^. If the putative HAO identified in the genome studied here functions as a true HAO, we speculate that it may catalyze the oxidation of hydroxylamine (NH_2_OH) to nitric oxide (NO) coupled to NO reduction via the NO reductase, with concomitant proton translocation in which the *bc*_*1*_ complex may also serve as an electron carriers and for protons translocation (Fig. 3).

Pathway(s) for electron flow during ß-oxidation of fatty acids (described above) may involve soluble electron transferring flavoproteins (ETFs) (identified in the genome next to the genes encoding for ß-oxidation) and/or membranous ETF–quinone oxidoreductase (ETF:QO), but ETF:QO was not identified^39^,^40^. It has been proposed that ETFs may transfer electrons to a membrane-bound FeS oxidoreductase to make H_2_ or formate^41^. Interestingly, a gene encoding a FeS oxidoreductase similar to the one identified in this prior study was found in the genome studied here, and it is co-located with the ß-oxidation genes, consistent with a role in receiving electrons from ETFs. In mitochondria, ETFs and ETF:QO link oxidation of fatty acids to the mitochondrial oxidative phosphorylation chain^39^. Thus, the configuration of the oxidative phosphorylation pathway indicates that the organism studied here may also be capable of NO reduction, likely linked to fatty acid oxidation.

We identified two NiFe hydrogenases in the genome. Phylogenetic analyses of the NiFe hydrogenase catalytic subunits revealed that the two NiFe hydrogenases are type 3b cytoplasmic hydrogenases most closely related to those of fermentative, sulfur-reducing Thermococcales Archaea, as previously reported in organisms from the Parcubacteria superphylum^6^. The type 3b hydrogenases may produce H_2_ during fermentation or H_2_S when polysulfide is available. Alternatively, they may consume H_2_ to produce the reduced form of nicotinamide adenine dinucleotide phosphate (NADPH) for anabolic metabolism^42^,^43^,^44^.

Given the phylogenetic and metabolic analyses presented above, we propose the name ‘Candidatus *Parcunitrobacter nitroensis*’ for the bacterium studied here, and suggest it to be a member the Parcunitrobacteria Class or Phylum (further sequences from this group are required to clearly resolve this placement). The name reflects the affiliation of the organism with the Parcubacteria and its unusual ability to carry out respiration linked to nitrogen compound metabolism. We suggest this lineage to be placed as Genus Parcunitrobacter, Family Parcunitrobacteraceae, Order Parcunitrobacteria and Class and/or Phylum Parcunitrobacteria within the superphylum Parcubacteria.

The sequences of all of the enzymes distinctive to ‘Candidatus *Parcunitrobacter nitroensis*’ are divergent from sequences found in other organisms, suggesting that these capacities were not recently acquired from non-CPR organisms (Table S1). Specifically, the percent of identity of the enzymes involved in respiration/TCA cycle to orthologs in non-CPR bacteria is low, ruling out recent lateral acquisition. Also, we did not detect the majority of the enzymes described above in other members of the CPR, consistent with the rarity of respiratory capacities across this group.

### Biosynthetic pathways

Although the catabolic potential of ‘Candidatus *Parcunitrobacter nitroensis*’ appears to be well developed, the genome analysis suggests limited anabolic machinery. We predict that this bacterium is not capable of biosynthesis of multiple amino acids as we find evidence only for the synthesis of glutamate and glutamine (and potentially aspartate). There is no indication of the ability to synthesize the cofactors riboflavin, folate, thiamine, nicotinate and nicotinamide, coenzyme A, pyridoxal phosphate. The genome encodes near-complete pathways for de novo biosynthesis of pyrimidine nucleotides but there are few enzymes for purine metabolism. Thus, we conclude that this organism may be auxotrophic for many essential metabolites or may contain novel biosynthetic pathways. The cell may obtain these compounds from its surroundings or through specific symbiotic relationships. The genome contains numerous proteases and peptidases, as well as several transporters, whose substrates are often unknown. We speculate that the predicted peptidases (Fig. 3) might generate oligopeptides and amino acids likely used to supplement auxotrophies, as an amino acid permease was identified in the genome.

### Cell surface, environmental interactions and defense mechanisms

The genome encodes 20 glycosyltransferases, which are essential for the biosynthesis of various saccharide molecules^45^ that mediate functions from structure and storage to signaling. The abundance of glycosyltransferases suggests that production of polysaccharides, glycoproteins and/or a glycosylated S-layer is important for this microorganism. The genome also encodes all four genes for the synthesis of deoxythymidine diphosphate (dTDP)-L-rhamnose (rmlA, rmlB, rmlC, and rmlD). dTDP-L rhamnose is the precursor of L-rhamnose, which is broadly found in plants and bacteria^46^. We identified all the genes involved in biosynthesis of peptidoglycan, but pathways for fatty acid, isoprenoid and S-layer biosynthesis are lacking. ‘Candidatus *Parcunitrobacter nitroensis*’ does not appear to make lipid A or lipopolysaccharide, as indicated by the absence of genes for their biosynthesis, including lpxC and kdsA^47^. Overall, and as for all CPR studied to date, the mechanism of formation and structure of its cell envelope remain unclear.

The genome is predicted to encode the necessary components for biosynthesis of type IV pilus, including pilT^48^ that confers twitching motility, and several pilins, as well as two type II secretion related genes. These type IV pili are not related to the sortase-associated pili more frequently found in gram-positive bacteria^49^. Instead, they might be similar to type IV pili involved in the uptake of environmental DNA^50^. Alternatively, the type IV pili may be involved in inter-organism interaction, as previously suggested^6^,^9^,^11^. We identified a polytopic membrane protein, ComEC, which is essential for DNA transport and competence as well as a DNA protection protein DprA. The DprA protein has been suggested to be involved in the protection of incoming DNA^51^.

While most CPR bacteria possess multiple copies of cell surface-associated proteins with one or more of the following domains: concanavalins/lectins, pectin lyases, fibronectin III, beta propeller, sortase motifs^9^,^33^, ‘Candidatus *Parcunitrobacter nitroensis*’ has few predicted enzymes with these domains. In fact, we identified only a few proteins predicted to have cell wall localization. These included proteins with beta- propeller and s-layer domains and proteins annotated as lipoproteins and involved in surface attachment (Fig. 3).

The genome encodes multidrug efflux systems and has key genes for protection from reactive oxygen species (ROS) including superoxide dismutase and thioredoxin-dependent peroxiredoxin, which could function as H_2_O_2_ scavengers. Thioredoxin reductase, which is important for the regeneration of reduced thioredoxin as a prerequisite for H_2_O_2_ detoxification by peroxiredoxins, was also identified in the genome. The genome also encodes for synthesis of the compatible solute trehalose. The disaccharide trehalose is widely distributed in nature^52^ and can protect the integrity of the cell against a variety of environmental challenges and serve as a carbon storage compound.

### ‘Candidatus *Parcunitrobacter nitroensis*’ proteins are highly divergent from those of other Parcubacteria

To examine how divergent the organism studied here is from its closest relatives, we determined the percent amino acid identity (AAI) between the proteins of this organism and those of the six mostly closest related Parcubacteria bacteria for which genomes were available (Table 1). The predicted proteins share no more than 50% AAI with those of its closest relatives from the Parcubacteria superphylum (Table 1A). However, none of the genomes in the comparison set share more than 70% AAI with other genomes in the comparison set, a finding that reflects that massive evolutionary scale of the Parcubacteria.

For the genomes used for comparative analysis, we determined the percentage of genes that have an ortholog in another genome. No more than 29% of ORFs in ‘Candidatus *Parcunitrobacter nitroensis*’ have orthologs in the comparison genome dataset (Table 1B). The 416 genes common between Parcunitrobacter and at least one of the six selected Parcubacteria genomes are involved in DNA repair functions, cell division, transcription, translation and oxidative stress. Also in this set are chaperons, type IV pili biogenesis proteins, two-rod shape-determining proteins, enzymes from glycolysis and the non-oxidative branch of the pentose phosphate pathway, oxoglutarate/2-oxoacid-ferredoxin oxidoreductase, the cytoplasmic type 3b hydrogenase, lactate dehydrogenase, multiple peptidases and glycosyl transferases (Table S2). These enzymes are commonly found in CPR genomes.

Of the ‘Candidatus *Parcunitrobacter nitroensis*’ 525 genes not found in any of the most closely related Parcubacteria genomes, 290 have no known function. Those with predicted functions are involved in energy production and conversion (including nitrogen metabolism and respiratory capacity described above), transport of metabolites, amino acids biosynthesis, biosynthesis of saccharide molecules and sugar interconversion (Table S2). Many of these functions are known to vary among even closely related species^53^.

## Conclusion

The Parcubacteria group of the CPR represents a vast amount of evolutionary divergence and thus it is considered to be a superphylum^12^,^15^. Despite this, the genomes of all previously studied Parcubacteria (and in fact most CPR) bacteria indicate very restricted metabolic potential, largely centered on fermentation. Thus, it is very interesting that ‘Candidatus *Parcunitrobacter nitroensis*’ has a genome that encodes versatile metabolic pathways, with the first complete electron transport chain described for a member of the CPR. It has respiratory and fermentative capacities as well as nitrogen and fatty acid metabolism. We predict that this unusual combination of metabolic features may be advantageous when viewed in the context of the metabolism of a (as yet unknown) specific host. Although the metabolic repertoire points to a different lifestyle compared to other CPR bacteria, Parcunitrobacteria still appears to depend on other organisms (or the community) for many basic building blocks. Specifically, Parcunitrobacteria appears to be unable to synthesize some essential metabolites, including amino acids, purine, lipids and cofactors. Many predicted ORFs were annotated only at the protein domain level or not at all, thus some unannotated proteins may complete metabolic pathways that appear to be absent. The large deviation in gene content of ‘Candidatus *Parcunitrobacter nitroensis*’ relative to all other CPR bacteria studied to date, as well as the lack of evidence for recent acquisition of its unusual respiratory and core metabolic capacities via lateral transfer, raises important questions about the evolutionary history of the CPR. Most intriguing of these may be the relative importance of genome reduction, lateral gene transfer, and shared ancestry in explaining the largely consistent, but now not fully consistent, metabolic profile across this massive radiation. The findings of this study suggest that additional metabolic peculiarities may be discovered in other bacteria of this radiation.

## Materials and Methods

### Sampling

As previously described, the field experiment was carried out between August 25 and December 12, 2011 at the Rifle Integrated Field Research Challenge (IFRC) site adjacent to the Colorado River, Colorado, USA^8^,^12^,^14^. In brief, acetate was added to groundwater to achieve an aquifer concentration of ~15 mM acetate in a manner consistent with previous such experiments at the site^54^. Acetate addition was terminated after 72 days. Biomass samples were collected prior to (sample GWA) and during (samples GWB, GWC, GWD, GWE) acetate addition, as well as after acetate addition ceased (sample GWF). Microbial cells pumped from groundwater that passed through a 1.2 μm pre-filter (Pall, NY) were retained on sequential 0.2-μm (designated 2) and 0.1 μm (designated 1) filters. Filters were flash-frozen in liquid nitrogen immediately upon collection for DNA extraction.

### DNA sequencing, assembly, binning, and annotation

As described previously^12^, DNA was extracted from the filters, sequencing libraries constructed, and 150 bp paired-end Illumina HiSeq 2000 sequences obtained. Sequence data were processed using the Illumina CASAVA pipeline (version 1.8). Reads were trimmed using Sickle (https://github.com/ najoshi/sickle) and assembled using IDBA-UD (-step 20, -maxk 100, -mink 40)^55^. Scaffolds were binned to genomes based on tetranucleotide and time-series abundance patterns using the ABAWACA algorithm (https://github.com/CK7/abawaca) and manually based on GC content, coverage, and taxonomic affiliation using ggKbase tools (http://ggkbase.berkeley.edu).

As part of this study, the draft genome for one bacterial population (GWA2_OD1-rel_38_13) reported by Brown *et al*.^12^ was subject to further curation. Multiple rounds of analysis involved sequential scaffold extension using reads mapped to the ends of scaffolds using either Geneious (http://www.geneious.com^56^) or Bowtie2^57^, with consideration of paired read distances. Local scaffolding errors were corrected or replaced with Ns using the script ra2.py (https://github.com/christophertbrown/fix_assembly_errors 12) and by manual curation that made use of unmapped read pairs. This approach also enabled filling scaffolding gaps and joining scaffolds. Genome binning was refined based on an Emergent Self-Organizing Map (ESOM) trained using time series abundance patterns and tetranucleotide frequencies, following well-established methods^58^,^59^. Reads from all metagenomes sequenced from 0.2 μm filters were used for determining abundance patterns in the ESOM analysis. Abundance data was weighted five times more than tetranucleotide frequencies because there were fewer samples used for calculating abundance patterns than tetranucleotides. ESOM training was conducted using the Somoclu^60^ algorithm with the option to initialize the codebook using PCA. Additional GWA2_OD1-rel_38_13 genome fragments were identified by finding scaffolds assembled from the GWA2 metagenome with similar abundance and tetranucleotide frequency patterns. A similarity cutoff was set as the maximum Euclidean distance between GWA2_OD1-rel_38_13 fragments and best matching neurons in the ESOM after removing the highest 2% of values. Then, the Euclidean distance between each GWA2 scaffold and each GWA2_OD1-rel_38_13 associated ESOM neuron was calculated. Scaffolds were added to the genome bin if they passed the abundance and tetranucleotide Euclidean distance threshold, and were associated with the GWA2_OD1-rel_38_13 bin in a subsequent ESOM analysis.

Protein-coding genes were predicted using Prodigal^61^, and 16S rRNA genes using SSU-Align^62^. The genomes were annotated by searching the predicted proteins against UniProt^63^, Uniref90^64^, KEGG^65^ and a custom in-house database^12^ using Usearch (-ublast)^66^. Orthologs were defined by reciprocal best usearch (-ublast) hits between proteins from each pair of genome sequences. Reciprocal best hits with E-value ≥ 0.01, bit score ≥ 40 and alignments covering ≥0.65 of the protein sequence were considered orthologs. For genome completeness and purity, we analyzed the presence or absence of single-copy genes (SCG) using 43 universal marker genes previously determined to be appropriate for CPR genomes^12^.

### Phylogenetic analysis

Phylogenetic analysis for validating the taxonomy of the genome described in this study was performed with the 16S ribosomal RNA (SSU) gene. A maximum-likelihood tree was constructed with RAxML-HPC under the GTRCAT model with 100 bootstrap re-samplings (RAXML CITATION). The 16S rRNA gene phylogeny was confirmed based on a previously performed analysis of 16 syntenic universal ribosomal proteins (RP) (L2, L3, L4, L5, L6, L14, L15, L16, L18, L22, L24 and S3, S8, S10, S17, S19)^15^.

For specific functional genes of interest, reference datasets were generated from sequences mined from NCBI databases. Alignments were generated using MUSCLE v.3.8.31^67^,^68^, curated manually, and phylogenies determined using PhyML^69^ under the LG + Gamma model of evolution with 100 bootstrap resamplings.

### Open-access database for the genome analysis

A summary of genome bin size, GC content, and coverage is located at http://ggkbase.berkeley.edu/Parcunitrobacteria/organisms/63175. All genomic information is publicly accessible via the website. We used the lists and genome summary functions to assess genome completeness and profile metabolic traits (http://ggkbase.berkeley.edu/genome_summaries/1088-Parcunitrobacteria_curated_genome). All fasta files for our figures are hosted on this site.

## Additional Information

**How to cite this article**: Castelle, C. J. *et al*. Unusual respiratory capacity and nitrogen metabolism in a Parcubacterium (OD1) of the Candidate Phyla Radiation. *Sci. Rep.* **7**, 40101; doi: 10.1038/srep40101 (2017).

**Publisher's note:** Springer Nature remains neutral with regard to jurisdictional claims in published maps and institutional affiliations.

## Supplementary Material

Supplementary Dataset 1

Supplementary Dataset 2

Supplementary Dataset 3

Supplementary Tables

## Figures and Tables

**Table 1:** Cross-genome comparison of Parcunitrobacteria and the six most closely related parcubacterial species.

	Parcunitrobacteria	OD1-1	OD1-2	OD1-3	OD1-4	OD1-5	OD1-6
**A**- Average percent amino acid identity for proteins encoded in the Parcunitrobacteria genome and the six most closely related Parcubacteria (OD1) genomes							
Parcunitrobacteria	—	48	47	47	50	49	47
OD1-1	48	—	58	52	51	52	58
OD1-2	47	58	—	53	51	52	59
OD1-3	47	52	53	—	52	52	52
OD1-4	50	51	51	52	—	70	51
OD1-5	49	52	52	52	70	—	51
OD1-6	47	58	59	52	51	52	—
**B**- Percentage of ORFs orthologous between pairs of Parcubacteria (OD1) genomes							
Parcunitrobacteria	—	29	17	18	29	28	27
OD1-1	35	—	22	24	36	32	37
OD1-2	38	40	—	29	38	32	38
OD1-3	38	43	27	—	41	36	38
OD1-4	39	40	22	25	—	49	36
OD1-5	37	35	19	22	48	—	33
OD1-6	32	37	20	21	32	30	—

(**A**) Percent amino acid identity among the analyzed OD1 genomes. **(B)** Percent (%) of ORFs that are orthologous for the OD1 genomes. **OD1-1**: GWB1_GWB1_OD1_42_6_43_6; **OD1-2**: GWF2_Parcubacteria_42_8; **OD1-3**: GWF2_Parcubacteria_bacterium_GW2011_GWF2_39_13b_39_13; **OD1-4**: RIFCSPHIGHO2_01_FULL_RIFCSPHIGHO2_01_FULL_RIF_OD1_08_40_12b_curated_40_12; **OD1-5**: RIFCSPLOWO2_01_FULL_RIFCSPLOWO2_01_FULL_RIF_OD1_08_38_39_curated_38_39; **OD1-6**: RIFCSPLOWO2_01_FULL_RIFCSPLOWO2_01_FULL_RIF_OD1_08_43_11_curated_43_11.
